# Editorial: Intersectional inequalities in work and employment: advances, challenges and renewed possibilities

**DOI:** 10.3389/fsoc.2024.1441849

**Published:** 2024-06-28

**Authors:** Jenny K. Rodriguez, Elisabeth Anna Guenther, Stella Nkomo, Marcela Mandiola

**Affiliations:** ^1^People, Management & Organizations Division, The University of Manchester, Manchester, United Kingdom; ^2^Centre for Teacher Education, University of Vienna, Vienna, Austria; ^3^Department of Human Resource Management, University of Pretoria, Pretoria, South Africa; ^4^17, Instituto de Estudios Críticos, Mexico City, Mexico

**Keywords:** intersectional inequalities, work and employment, multiple identities, privilege, disadvantage

Intersectionality is one of the most important theoretical, methodological, and analytical tools to explore inequalities in social and economic life and understand how power relations are sustained between groups of people (Collins, 2000; Holvino, 2010). Its coinage in 1989 (see Crenshaw, 1989) is preceded by discussions in Black Feminism about the complexities linked to the lived experiences of disadvantage of Black women and women of color (see Collins, 2000; Hancock, 2016) and advanced by discussions that interrogate the ways race alongside other intersecting multiple identities are implicated in the organization of social and economic life (see Rodriguez et al., 2016; Nkomo, 2021). Theoretically, intersectionality sees multiple identities as mutually constitutive, overlapping, and interdependent, explaining how they interplay within interlocked systems of oppression to create instances of privilege and oppression. Intersectionality's engagement with the complexity of multiple identities means that it “adds the specificity of sex and gender to race and ethnicity, and racial and ethnic specificity to sex and gender” (MacKinnon, 2013, p. 1020). Methodologically, intersectionality enables the exploration of the points of convergence of multiple identity systems, focusing on the outcomes and structural realities that shape these points, and facilitating the articulation of more nuanced and complex stories of privilege and disadvantage (Crenshaw, 1991; Collins, 2019; Haynes et al., 2020). Analytically, it enables a nuanced understanding of what produces and perpetuates domination; Cho et al. (2013) note the importance of developing an analytical sensibility that emphasizes what intersectionality does, rather than what it is. In this respect, as an analytical tool, intersectionality is generally connected to a critical interrogation of power dynamics with a view to understand how these can be challenged and eliminated.

Despite the significant advances in embedding intersectional lenses to the analysis of disadvantage, there is scope for more interrogation of Intersectional inequalities. In particular, the strength of intersectionality's theoretical foundations to explain privilege and disadvantage remains stronger than its empirical and practical uses to inform transformational change and social justice. A key argument put forward by intersectional thinking is that it is not enough to look at racialized and gendered practices separately. Instead, it is important to discuss the simultaneity of different forms of oppression. When Crenshaw (1989) introduced intersectionality, she emphasized the importance of the simultaneous interplay of race and gender to grasp existing forms of inequalities. However, simultaneity within work is not limited to the interplay of social categories. As Holvino (2010) argues, it is important to also examine the simultaneous processes of identity, institutional and social practice through linking societal and organizational processes. Another layer of simultaneity comes with the interrogation of privilege and disadvantage, as this helps us to understand that due to the multiple forms of oppression people can carry both privileges and disadvantages (Collins, 2000) and that the reading of these might differ depending on the context (Rodriguez and Ridgway, 2019). In the context of new, emerging, and re-configured inequalities affecting workers, workforces, workplaces, and the future of work, it is important to continue utilizing exploring these issues with intersectionality and developing intersectional analyses to interrogate understandings of multiple identities, how they are connected to inequalities, and how they shape experiences of work and employment.

Intersectionality has traveled to the field of work and employment. Rodriguez (2024) notes that there is a trajectory of engagement with intersectionality, where its adoption has followed three distinguishable trends as a framework to understand the interplay of multiple social categories with work and employment experiences and outcomes for individuals, in organizations and more widely in labor markets: (1) Works focusing on experiences of groups located at the point of multiple disadvantages (e.g., Opara et al., 2020; Adapa and Sheridan, 2021; Sliwa et al., 2023), (2) Works focusing on individual and organizational outcome differentials for groups located at the point of multiple disadvantages (e.g., Cech and Rothwell, 2020; Netto et al., 2020; Kele et al., 2022), and (3) Works focusing on labor market level analyses of outcome differentials for groups located at the point of multiple disadvantages (e.g., Yemane, 2020; Berghs and Dyson, 2022; Kim and Lee, 2023). These works have helped to advance a better grasp of intersectional dynamics within work and organizations, contributing to an understanding of multiple forms of power and their consequences for inequalities.

## Contributions to the Research Topic

This Research Topic brings together five papers that engage, in different and interdisciplinary ways, with the call for advances, challenges and renewed possibilities of intersectional dimensions related to work and employment, presenting important and innovative research currently being undertaken in the field in different countries, namely the UK, the Czech Republic, Serbia, Portugal and New Zealand.

The first paper is “*Fitting in whilst standing out': Identity flexing strategies of professional British women of African, Asian, and Caribbean ethnicities*” by Opara et al. Drawing on literature on intersectional identity work and identity management strategies, the authors explore the workplace experiences and challenges faced by 30 professional British women of African, Asian, and Caribbean (AAC) ethnicities. The paper unveils ACC women's identity flexing strategies, identifying four themes: (1) the benefits of identity flexing, (2) the role of specific stereotypes, (3) context specific opportunities, and (4) the costs of identity flexing. The paper's findings show that identity flexing is deployed in a situated manner to mobilize identity features that are more valued within social contexts. This suggests that identity flexing enables the women to agentically navigate workplace environments in order to control the narratives about them, adapt to settings and manage stereotypes. Conversely, the study also identifies that these efforts involve much emotional labor and can lead to feelings of inauthenticity.

The second paper is “*Determinants of individual income in EU countries: implications for social policy targeting*” by Baláková et al. In this paper, the authors introduce the Income Index, which enables the analysis of individual data from all EU countries to gain insight into the factors that influence income and shape inequality. Drawing on microdata from the EU-SILC survey (European Union—Statistics on Income and Living Conditions), specifically, the EU-SILC 2021, the authors develop multiple hierarchical regression and show that gender, age and highest attained level of education are implicated in income inequality. More generally, the study shows that household composition, occupation sector and the degree of urbanization are the factors that most significantly affect income inequality. The paper reinforces the disadvantageous position of single parent households in the labor market and their need for social support. The paper provides evidence that can inform the development of social policies.

The third paper is “*A discussion of gender, ethnicity, and intersectionality, at the Serb Business Association forum*” by Paravina. This paper is an example on the complex, situated interplay of racialized and gendered processes. The author analyzes public discussions of Serb women living in Croatia and the stigmatizing and ostracizing practices they perceive as members of an ethnic minority. Drawing on Conversation Analysis and Membership Categorization Analysis the author highlights barriers ethnic minority women face when they want to invoke institutional rules related to their human rights. The paper contributes to an intersectional understanding of institutional rules, where it brings important points and tensions from a reading of ethnicity and gender rights, the contextualization of ethnicity in a personalized manner, a downgrading of ethnicity rights through reformulation; and the use of laughter as a means to show ambivalence toward gender equality and ethnicity.

The fourth paper is “*Working conditions and attitudes toward work: the case of Portuguese youth from Braga*” by Duque and Vázquez. Drawing on discussions about work precariousness and unstable work trajectories, the paper explores the working conditions and attitudes to work among youth people in Braga, Portugal. Using survey data gathered from a sample of 406 young people, the findings suggest an important generational shift characterized by the lack of centrality of work as a defining aspect of their identity. The paper mobilizes the idea that young people have a dense social experience that is shaped by work-related factors (e.g., task performed and contractual conditions) as well as broader factors (e.g., economic, social and cultural aspects). The paper highlights that, rather than to work itself, young people's identities are more strongly linked to the sociability that work enables and to other life spheres where they seek fulfillment.

The fifth paper is ““*It made me feel like a shit parent”: an intersectional analysis of pandemic mothering*” by Thorpe et al. The paper focuses on the maternal experiences of Māori, Pacific, Asian and migrant mothers living in Aotearoa New Zealand during the COVID-19 pandemic. The study adopted an intersectional lens to analyze data gathered from a sample of 24 mothers (including women who were pregnant and gave birth during lockdowns, teenage mothers, single and low-income mothers, and working mothers). Findings suggest that mothers faced challenges based on their diverse social, cultural, and economic positionalities, in some cases intensified due to isolation, judgment, and discrimination associated with intersectional disadvantages. It is also highlighted that the pandemic affected how the women felt about motherhood and reframed their relationships with home, family, work, and broader society. The paper contributes to an understanding of the gendering of everyday maternal life, intersectional inequalities and identifies the need for more intersectional culturally and gender-responsive policies that tackle the multi-layered complexities of mothers' lives.

## Conclusion: a call for renewed possibilities and new/reformulated questions

The present socio-cultural and political moment is characterized by conflicting discourses that, while recognizing the importance of taking action to tackle grand challenges such as social justice, also promote post-racial, post-identitarian discourses that undermine efforts to achieve social justice. There are several areas where the theoretical, methodological, and analytical potential of an intersectional lens can shed light into the discussion about work and employment dynamics. For instance, intersectionality can support the interrogation of the complexity of hierarchies of power and how these hierarchies play out in dynamics of work and employment. Intersectional scholars have called for more emphasis on the entanglement of different domains or layers; for instance, Thatcher et al. (2023) call for the acknowledgment of power structures at societal, organizational, interpersonal, and individual levels, suggesting that a multilevel approach is essential to grasp the functions, effects and origins of different forms of inequalities.

Moving forward, we must rethink the questions we ask to meaningfully advance discussion in ways that capitalize on intersectionality's emancipatory potential to support and develop equitable and sustainable futures for workers and workplaces. In critically reflecting about how intersectionality has traveled and advanced in the field of work and employment, there are three distinct areas where more scholarly work needs to be developed in order to advance discussions about intersectional inequalities: Clearer engagement with structural/institutional dimensions of intersectional inequalities, developing integrated intersectional frameworks that move beyond the gender+ approach, and a more nuanced interrogation of power and privilege. The previous points need to be positioned within the wider call to develop intersectional work that challenges the post-identitarian, dis-identity or identity-skeptical theoretical milieu and reclaiming the analytical spaces that expose racism, patriarchy, heterosexism, ableism and classism and challenge oppressive power and privilege in work and employment. In addition, more methodological and empirical innovation is needed to develop situated intersectional frameworks that recognize contextual specificities and engage with the reconfigured and new inequalities emerging from discussions about the future of work, such as the temporary/transformative change continuum (Schwartz, 2021), technological transformation (Trenerry et al., 2021), and the ‘new possible' (Emmett et al., 2021).

## Author contributions

JR: Writing – original draft, Writing – review & editing. EG: Writing – original draft, Writing – review & editing. SN: Writing – original draft, Writing – review & editing. MM: Writing – original draft, Writing – review & editing.
